# Retrospective Analysis and Forecasted Economic Impact of a Virtual Cardiac Rehabilitation Program in a Third-Party Payer Environment

**DOI:** 10.3389/fdgth.2021.678009

**Published:** 2021-11-24

**Authors:** Arash Harzand, Aaron C. Weidman, Kenneth R. Rayl, Adelanwa Adesanya, Ericka Holmstrand, Nicole Fitzpatrick, Harshvardhan Vathsangam, Srinivas Murali

**Affiliations:** ^1^Emory University School of Medicine, Atlanta, GA, United States; ^2^VITAL Innovation, Highmark Health, Pittsburgh, PA, United States; ^3^Moving Analytics, Inc., Los Angeles, CA, United States; ^4^Cardiovascular Institute, Allegheny Health Network, Pittsburgh, PA, United States

**Keywords:** mobile health, virtual care, cardiac rehabilitation, economic impact, coronary artery disease

## Abstract

**Background:** Participation in cardiac rehabilitation (CR) is recommended for all patients with coronary artery disease (CAD) following hospitalization for acute coronary syndrome or stenting. Yet, few patients participate due to the inconvenience and high cost of attending a facility-based program, factors which have been magnified during the ongoing COVID pandemic. Based on a retrospective analysis of CR utilization and cost in a third-party payer environment, we forecasted the potential clinical and economic benefits of delivering a home-based, virtual CR program, with the goal of guiding future implementation efforts to expand CR access.

**Methods:** We performed a retrospective cohort study using insurance claims data from a large, third-party payer in the state of Pennsylvania. Primary diagnostic and procedural codes were used to identify patients admitted for CAD between October 1, 2016, and September 30, 2018. Rates of enrollment in facility-based CR, as well as all-cause and cardiovascular hospital readmission and associated costs, were calculated during the 12-months following discharge.

**Results:** Only 37% of the 7,264 identified eligible insured patients enrolled in a facility-based CR program within 12 months, incurring a mean delivery cost of $2,922 per participating patient. The 12-month all-cause readmission rate among these patients was 24%, compared to 31% among patients who did not participate in CR. Furthermore, among those readmitted, CR patients were readmitted less frequently than non-CR patients within this time period. The average per-patient cost from hospital readmissions was $30,814 per annum. Based on these trends, we forecasted that adoption of virtual CR among patients who previously declined CR would result in an annual cost savings between $1 and $9 million in the third-party healthcare system from a combination of increased overall CR enrollment and fewer hospital readmissions among new HBCR participants.

**Conclusions:** Among insured patients eligible for CR in a third-party payer environment, implementation of a home-based virtual CR program is forecasted to yield significant cost savings through a combination of increased CR participation and a consequent reduction in downstream healthcare utilization.

## Introduction

Cardiac rehabilitation (CR) is recommended for patients with coronary artery disease (CAD) to reduce the risk of hospital readmission and cardiovascular (CV) death after an acute myocardial infarction (MI) or coronary procedure (1, 2). Despite the benefits, fewer than 20% of eligible patients enroll in center-based CR (CBCR) (3). Limited program availability, distance, and high cost make attending CBCR—typically delivered over 12 weeks with thrice weekly sessions—burdensome to patients and these factors all contribute to low participation (4). There is a clear need to develop effective and patient-centric alternatives to expand CR access for eligible patients (4). This need has only been magnified by the emergence of new safety considerations for higher-risk patients from travel and social exposure as well as the need for many CBCR programs to operate on more restricted schedules and reduced patient appointments during the ongoing COVID pandemic.

One proposed alternative is virtual, home-based cardiac rehabilitation (HBCR) which combines self-led exercise training with health coaching and remote patient monitoring, often through a mobile health platform (5). HBCR has been shown to be non-inferior to facility-based CR in large meta-analyses and is supported by clinical practice guidelines with a Class IIa (i.e., reasonable alternative) recommendation for patients who are unable or unwilling to participate in a facility-based program (6–8). Aside from fully integrated, risk-bearing healthcare systems such as Kaiser Permanente (KP) and the Department of Veterans Affairs (VA), widespread adoption of HBCR has remained poor among systems operating within third-party payer environments due to limited reimbursement (5, 6, 9–11).

**Figure 1:**
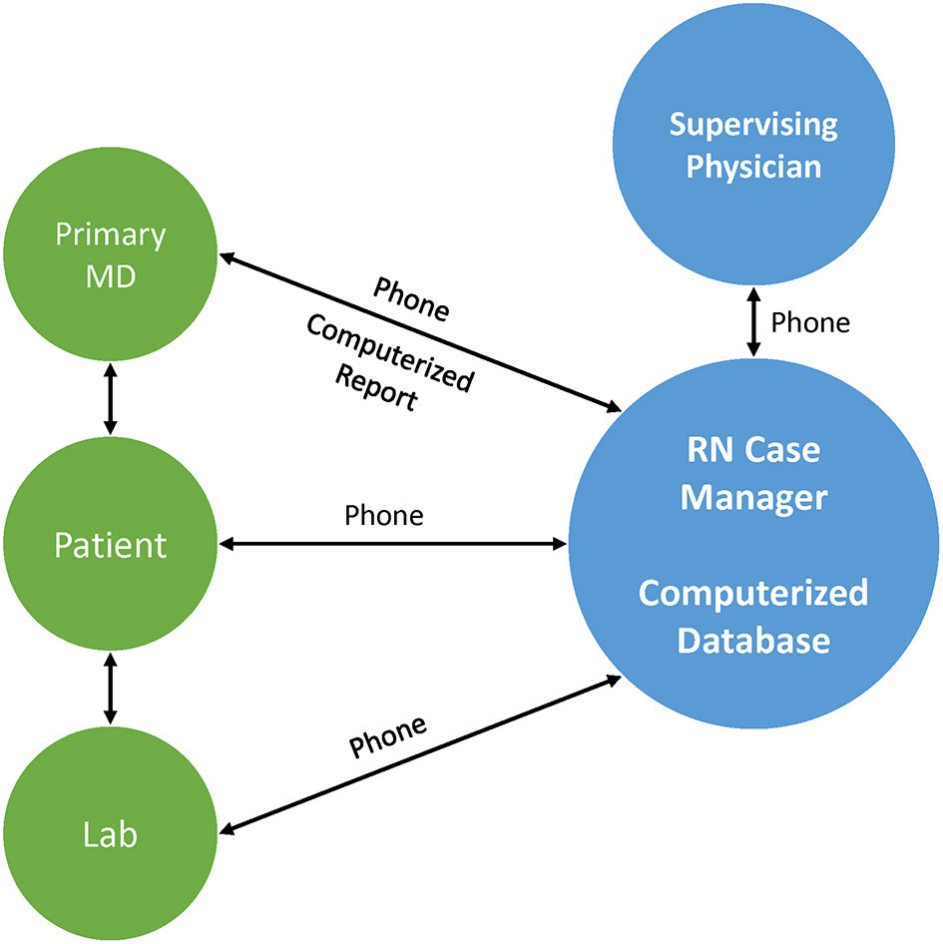
The MULTIFIT model of home-based cardiac rehabilitation.

MULTIFIT is an evidenced-based HBCR model with demonstrated success in reducing readmissions and adverse events in CAD patients and this program has been widely adopted among integrated care networks such as KP (Figure 1) (9, 10). MULTIFIT is a 12-week, nurse-mediated case-management program that delivers guideline-based recommendations for comprehensive CAD risk factor modification through remote encounters (12). During each virtual visit, a nurse manager provides counseling on and sets goals for exercise, smoking cessation and dietary modification using patient-derived algorithms that have been previously described (9). Among CAD patients within KP of Northern California, enrollment in MULTIFIT led to significant reductions in hospital readmission (49%), recurrent MI (58%), and all-cause mortality (53%) (13). More recently, MULTIFIT has been successfully implemented within the VA through a mobile health platform with high retention (90% at 30 days, 62% at 90 days) and high patient satisfaction (80%) (5). Still, the feasibility of delivering MULTIFIT as a virtual CR program within commercial payer environment remains unknown. Here, we evaluated the potential clinical and economic feasibility of implementing a virtual HBCR program based on MULTIFIT in a third-party payer system.

## Methods

Among eligible patients admitted for a CAD related diagnosis or procedure, our objectives in this retrospective cohort study were to (1) describe the current rates of CBCR enrollment and hospital readmission within 12 months after discharge, (2) calculate healthcare delivery costs including CBCR delivery, all-cause and CV related readmission, and (3) estimate the economic impact of implementing a MULTIFIT-based virtual HBCR program within a third-party payer system. For the economic feasibility calculation, we hypothesized that virtual HBCR adoption would generate economic savings through a combination of increased CR adoption from new patients that would have not otherwise participated in CR and subsequent downstream savings from reduced hospitalizations. The additional *cost* for new HBCR participants would therefore be outweighed by the *savings* that would result from improved downstream clinical outcomes.

### Data Sources

We retrospectively analyzed data from Highmark Health, a non-profit health care organization providing insurance coverage primarily throughout Pennsylvania, West Virginia, and Delaware. Claims data were derived from commercial, Medicare Advantage, and Medicare Supplemental insurance plans. Costs included in this report refers to allowed amounts, or the maximum possible payment that the insurance plan could be obligated to make to medical providers for services rendered.

### Population

Our population of interest included patients aged 18+ years with an index hospitalization for a CAD-related primary diagnostic or procedural code (e.g., CABG or PCI) between October 1, 2016, and September 30, 2017 (see Supplementary Table 1 for full list of codes). Patient insurance claims were examined post-discharge for an additional 12-months (through at latest September 30, 2018) to measure CR enrollment and readmission rates. We included all members who met eligibility criteria regardless of their primary state of residence; most Highmark members live in Pennsylvania, Delaware, and West Virginia, but members span all 50 US states. We only included patients with continuous enrollment in an eligible insurance plan for the 12 months following discharge to obtain complete follow up information. Federal Employee Program (FEP) members were excluded due to limitations in use of their claims data.

### CR Participation Rates

CR participation rate was defined as the percent of patients who had at least one insurance claim for an outpatient CR session during the 12-months follow discharging. For each patient, we also calculated the days between discharge and the initial CR session (CR enrollment lag) and the total number of CR sessions attended. We used *z*-tests to compare CR participation rates across subgroups (e.g., men vs. women).

### Healthcare Utilization and Hospital Readmission Rates

All-cause and CV-related readmission rates were calculated at monthly intervals up to 12-months post-discharge. We defined each hospital readmission by the presence of at least one inpatient insurance claim using billing codes indicating claim type (e.g., inpatient, outpatient) and place of service (e.g., hospital inpatient, urgent, or emergency care); emergency department and observation admissions were excluded. We identified CV-related encounters using “Major Diagnostic Category” codes in the insurer database. We used *z*-tests to compare the cumulative proportion of CR participants and non-participants who experienced hospital readmission at each monthly interval. We used *t*-tests to compare the mean number of hospital readmission episodes among patients who were readmitted at least once between the CR participant and non-participant groups.

### CR and Healthcare Costs

Per-patient healthcare costs for CBCR delivery were calculated from claims data. Per-patient HBCR cost was set at $1,550, based on the commercial price of the Movn platform (Moving Analytics, Los Angeles, CA; note that this value is akin to an allowed amount from an insurance perspective—it is the maximum amount the insurer would typically have to pay for the medical service—and is therefore comparable to our calculated cost of CBCR). To provide an initial check on generalizability for CR costs, we obtained national CBCR costs from claims incurred between January 1, 2017, and December 31, 2018, using the Blue Health Intelligence (BHI®) National Data Warehouse Analytical DataMart (ADaM), a database of insurance claims populated with contributions from private payers within the Blue Cross Blue Shield Insurance Network across all 50 US states. Episodic costs for all-cause and CV-related readmissions during the 12-month follow up period were also calculated using claims data.

### Clinical and Economic Forecasts

We conducted a clinical and economic analysis of HBCR implementation by forecasting scenarios in which: (a) some of the patients who did not originally participate in CBCR chose to participate via HBCR, and (b) this group of patients showed a lower rate of hospital readmission (14). We used previously published MULTIFIT data as a guide to define these scenarios and hypothesized a net savings as a result of (a) and (b) (12, 15, 16). We further conducted sensitivity analyses to examine the robustness of these forecasts to potential fluctuations in per-patient hospital readmission costs based on the data observed in the current study.

### Study Design and Oversight

Moving Analytics provided funding for the study through a contract with the VITAL Innovation Program at Highmark Health. The study was designed by Highmark Health in consultation with the sponsor. Highmark Health had unrestricted access to the study data and was responsible for data collection and analysis. Data were analyzed by two authors who are employed by Highmark Health (AW and KR). The sponsor and its affiliated authors (AH, AA, & HV) did not have access to the study data and participated in data interpretation only. The first and second authors (AH and AW) drafted the manuscript. The trial was approved by the IRB at Highmark Health and informed consent was waived for the retrospective claims analysis.

## Results

### CR Participation and Delivery Costs

**Figure 2:**
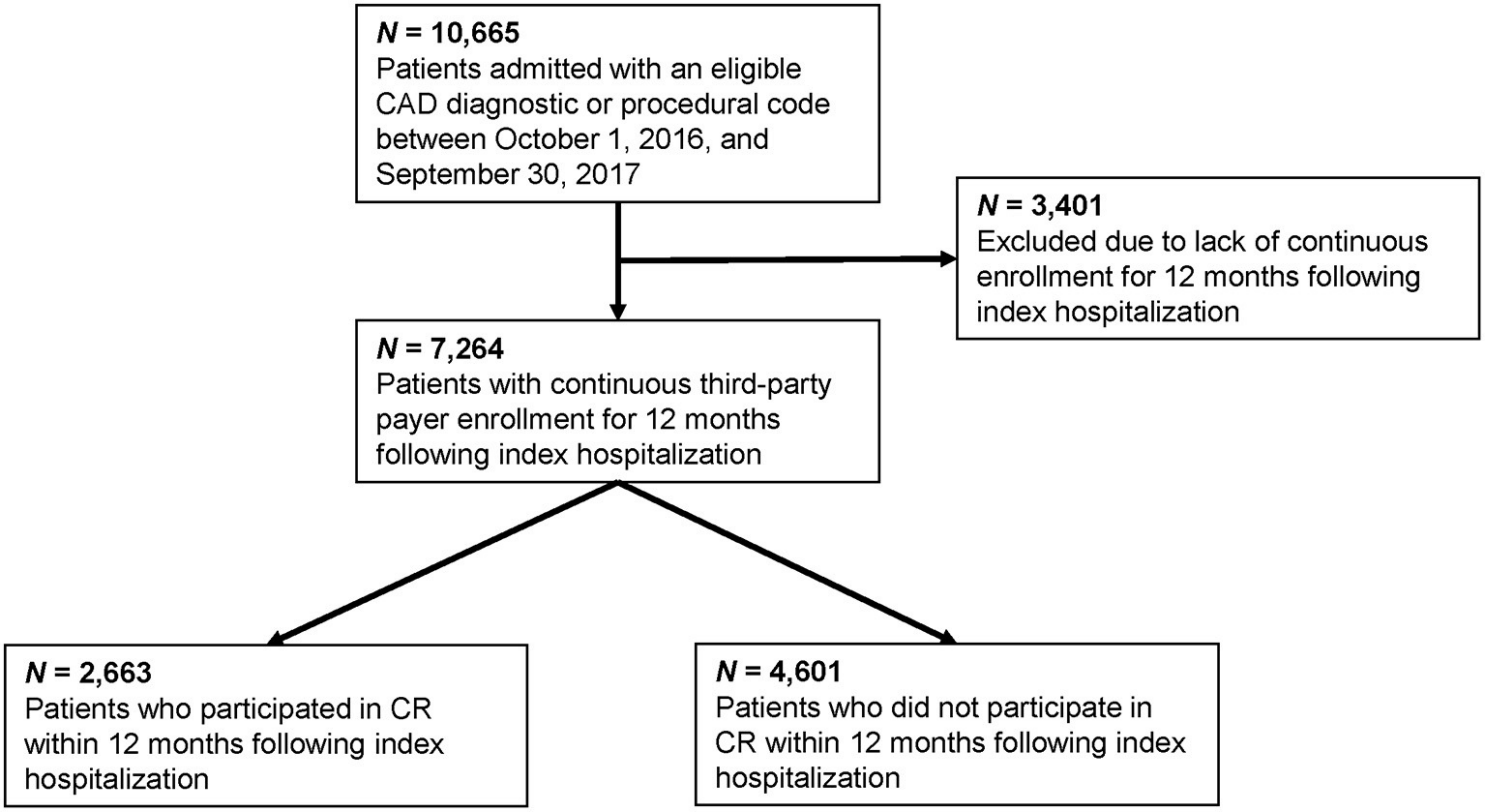
Patient flowchart.

Figure 2 displays a flowchart for patient identification and CR participation. We identified 7,264 unique inpatients who were eligible for CR (mean age = 67 ± 12.9 years; 67% men). CR participation was only 37% within 12 months of discharge (*n* = 2,663) with a mean enrollment lag of 49 ± 49 days. CR participants attended a mean of 23 ± 13 sessions with a mean per-patient allowed cost of $2,922 ± $2,413. Comparatively, data from BHI® ADaM demonstrated a mean annual cost of $2,870 per patient-member, speaking to the generalizability of our observed mean CBCR delivery cost.

Among subgroups, men participated at a higher rate than women (40 vs. 29%; *z* = 9.37; *p* < 0.001). CR participation was higher among patients <60 years of age (43 vs. 34% in those >60 years; *z* = 7.04; *p* < 0.001); consistent with this finding, participation was also higher in patients with commercial compared to senior-oriented (e.g., Medicare Advantage) insurance plans (42 vs. 31%; *z* = 9.42; *p* < 0.001; see Supplementary Tables 2, 3).

### Readmission Rates and Cost

**Figure 3:**
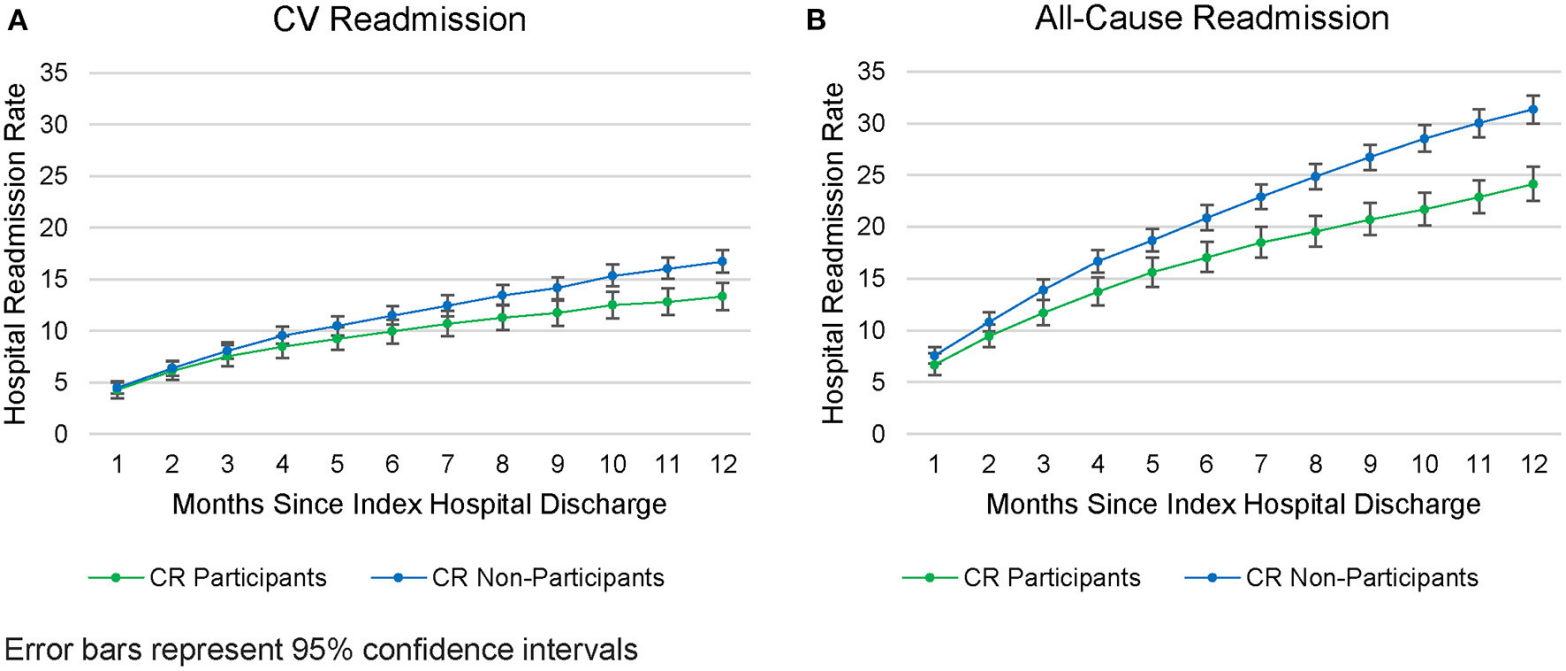
Readmissions rates from the CAD population. **(A)** CV Readmission. **(B)** All-cause Readmission.

Cardiovascular-related readmission rates were lower among CR participants than non-participants beginning at 6-months post-discharge (11.5 vs. 10%; *z* = 2.01, *p* = 0.045; see Figure 3A), a trend which continued until the 12-month time point (13 vs. 17%, *z* = 3.84, *p* < 0.001). An even earlier divergence was seen for all-cause readmission rates, which were lower among CR participants (vs. non-participants) beginning at 3-months post-discharge (11.7 vs. 13.9%; *z* = 2.70, *p* = 0.007; see Figure 3B) a difference that persisted until 12-months (24 vs. 31%, *z* = 6.61, *p* < 0.001). Furthermore, among patients who were readmitted, CR participants were readmitted less frequently than non-participants [1.48 ± 0.95 vs. 1.74 ± 0.35 readmissions; *t*(2, 084) = 4.42; *p* < 0.001; see Supplementary Table 4 for the most frequent readmission etiologies]. The mean per-patient, per readmission allowed amount did not differ significantly between the CR and non-CR groups [$32,164 vs. $30,213; *t*(2, 084) = 0.61; *p* = 0.55].

### Economic Analysis

HBCR would offer a viable option for the 63% of patients in our sample who declined facility-based CR. If some patients were to take this option, overall CR participation rate would increase, and hospital readmissions and associated costs would likely decline. We projected the economic impact of offering HBCR in a third-party payer population by modeling nine hypothetical scenarios which varied on: (a) the rate at which the 4,601 patients who previously declined CR enrolled in HBCR, and (b) the hospital readmission rate among this group of new HBCR participants.

To best anchor our economic forecasts for the Movn virtual program, we primarily relied on published data from prior MULTIFIT trials which represented the evidence base upon which Movn was developed. For HBCR enrollment, the “Pessimistic” scenario was set to reflect extremely low uptake (i.e., 10%), as this would imply a nearly complete rejection of the hypothesis that Movn would significantly increase enrollments. Subsequently, we set the “Conservative” scenario to closely match the lowest reported enrollment rate from prior MULTIFIT studies [41% from Levin et al. (16), and the “Optimistic” scenario was set to closely match the highest reported enrollment rate in prior MULTIFIT studies (80% in Landis et al. and 83% in DeBusk et al.)] (12, 15).

We applied similar rationale to determine our projected hospital readmission rates, again basing these values on the current state of readmissions at Highmark as well as previously published data from MULTIFIT. For each of the 3 scenarios (Pessimistic, Conservative, Optimistic) we performed 3 separate projections for possible readmission rates set to “Low,” “Moderate,” and “High.” We set the most pessimistic possible outcome (i.e., “High” rate of readmissions) to closely match the current state of hospital readmissions following center-based CR enrollment at Highmark (i.e., 24% based on our current data). The “Low” readmission rate was set to closely match the best/lowest reported readmission rate – we were limited with the existing published MULTIFIT studies as none previously reported on readmissions rates, hence we relied on published rates from the 2015 Cochrane review (which is now updated to the 2017 version by Anderson et al.). In both systematic reviews, the lowest reported readmission rate following an HBCR intervention was 8% (from Jolly 2007) which we rounded up to 10% for the “Low” readmission rate projection. The “Moderate” readmission rate projection was set to a value in between the “Low” and “High” estimates (i.e., 17%) (14, 17).

For each of the nine scenarios (3 CR participation rates^*^3 readmission rates), we compared the *projected* annual cost to the *current* annual medical cost of $72.1 million in our sample, which included (a) $28.5 million for delivery of facility-based CR to 2,663 patients and (b) $43.6 million for hospital readmission-related medical treatment for 2,087 patients. A projected cost that fell below $72.1 million would indicate a projected net savings under HBCR implementation.

We projected a median savings of $4.5 million across scenarios (see **Table 2**). Projected net savings varied considerably, increasing as the rate of HBCR increased and as the 12-month hospital readmission rate decreased. For example, at 10% HBCR participation and 24% readmission rate (identical to the current observed rate of 24%), projected savings were just $98,000, whereas projected savings were $17.3 million at 80% participation and 10% readmission rate.

### Sensitivity Analyses

Episodic hospital readmission costs showed considerable variability across patients, which could affect the result of our economic analysis (see Table 1). As a sensitivity analysis, we therefore ran two additional economic forecasts to account for potential fluctuation in hospital readmission cost in future cardiac care samples and/or in other payer environments. Under a “best case” economic outcome, we set the per patient readmission cost at $28,787 for CR patients (i.e., the low end of the 95% confidence interval for this value) and at $34,151 for non-CR patients (i.e., the high-end of the 95% confidence interval). Not surprisingly, this scenario yielded healthy projected annual savings, with a median of 7.9 million, never falling below $1.0 million, and ranging as high as $23.1 million (see Table 2).

**Table 1:** Variables used in economic impact analysis.

**Category**	**Scenario 1** **(Pessimistic)**	**Scenario 2** **(Conservative)**	**Scenario 3** **(Optimistic)**
HBCR CR participation rate	10%	40%	80%
**Patients**			
Facility-based CR	2,663	2,663	2,663
HBCR	460	1,840	3,681
Non-CR	4,141	2,761	920
**CR cost**			
Facility-based	← $2,922 [$2,830,$3,013] →		
HBCR	← $1,550 [Fixed] →		
**Readmission cost**			
CR Patients	← $32,164 [$28,787, $35,540] →		
Non-CR Patients	← $30,213 [$26,276, $34,151] →		
**Readmission rate**			
Low	10%	10%	10%
Moderate	17%	17%	17%
High	24%	24%	24%

*Cost values are mean [95% Confidence Interval].*

*← → indicates that a value is constant across all three scenarios*.

**Table 2:** Results of economic impact analysis.

**Category**	**Scenario 1 ** **(Pessimistic)**	**Scenario 2 ** **(Conservative)**	**Scenario 3 ** **(Optimistic)**
HBCR participation rate (anticipated)	10%	40%	80%
**Projected annual cost**			
Facility-based CR patients	← $28.4 →		
Non-CR patients	$39.3	$26.2	$8.7
**HBCR patients**			
**Readmission rate**			
10%	$1.5	$8.8	$17.5
17%	$3.2	$12.9	$25.8
24%	$4.3	$17.1	$34.1
**Projected total annual cost**			
**Readmission rate**			
10%	$69.2	$63.4	$54.7
17%	$70.9	$67.5	$63.0
24%	$72.0	$71.7	$71.3
**Projected net savings**			
**Readmission rate**			
10%	$2.8 [$2.1, $3.6]	$8.7 [$5.8, $11.6]	$17.3 [$11.6, $23.1]
17%	$1.1 [$0.3, $2.0]	$4.5 [$1.2, $7.9]	$9.0 [$2.4, $15.7]
24%	$0.01 [$ −0.08, $1.0]	$0.4 [$-3.4, $4.2]	$0.8 [$-6.7, $8.3]

*Projected costs/savings are in millions of dollars. Projected net savings compares the projected total annual cost under each modeled scenario to the current total annual cost of $72.1 million. 95% confidence intervals for projected net savings are taken from the above sensitivity analysis*.

Conversely, under a “worst case” economic outcome, we set the per-patient readmission costs at $35,540 for CR patients and $26,276 for non-CR patients (i.e., the high- and low-ends of the 95% confidence intervals, respectively). This scenario yielded a more mixed financial picture. At 10 and 17% readmission rates, we still projected savings that were often substantial (range: $300,000–11.6 million). In contrast, at 24% readmission rate, we projected a net *cost* to the healthcare system (range: $ –$800,000 to –$6.7 million; see Table 2). Note that the projected net cost at 24% readmission rate became *larger* with higher participation in HBCR, because HBCR patients in this scenario would incur costs both to pay for their cardiac rehab and to cover costs for their relatively high hospital readmission rate.

## Discussion

Our study shows that multidisciplinary CR remains significantly underutilized in population of 7,264 commercially and Medicare-insured beneficiaries with a CAD-related index hospitalization within a large third-party payer environment. Notably, the observed participation rate of 37% falls far below national initiatives to achieve ≥70% participation by 2022 (18), suggesting that inventive efforts, such as virtual HBCR, are needed to increase participation. Even if facility-based programs become increasingly available, ongoing concerns regarding CAD patients' safety during the COVID pandemic may continue to restrict availability (19).

We also forecasted the impact of offering HBCR to the 4,601 patients in our sample who originally *did not* participate in facility-based CR, by modeling (a) the rate at which the 4,601 patients who previously declined CR choose to participate in HBCR, and (b) the hospital readmission rate among this group of new HBCR participants. We projected a median annual savings of $4.5 million through a combination of increased CR participation and reduced hospital readmissions among new HBCR participants. These modal projections reflect the most plausible assumptions of a 40% HBCR participation rate and a 12-month hospital readmission rate for HBCR participants of 17% (see Table 2). Sensitivity analyses suggested that savings ranging from $98,000 to $17.3 million may theoretically be seen depending on local variations in HBCR enrollment and readmission as seen in prior studies (12, 14–16). Conversely, sensitivity analyses also helped to identify a boundary condition to the projected net savings, namely if the readmission rate among HBCR participants remains at or above our observed rate of 24% among CBCR participants, and if per-episode costs hospital readmission are higher than expected among all CR participants.

One benefit of HBCR therefore is cost-effectiveness, given that home-based programs provide a relatively inexpensive alternative to facility-based care ($1,550 vs. an average of $2,922 per-patient in our analysis). Several previous trials have shown a reduction in cardiac morbidity and hospital readmission as a result of facility-based CR participation (20, 21). Yet, these positive clinical outcomes can be partially offset by high investment and capital expense required for personnel, equipment, and space, thereby preventing the health system from realizing maximal benefits of traditional CR across large populations (22).

Another potential benefit of HBCR is a reduction in enrollment wait times as delays in enrollment are associated with mitigated benefits of CR following acute MI and CABG surgery (23, 24). Prior studies have shown that for every day that passes after hospital discharge, there is a ~1% decrease in CR participation (5). Among the factors associated with longer wait times include being employed and longer drive times to CR, both of which reduce the convenience of participation (24). Early enrollment within 21 days of a qualifying event is therefore an important quality metric that results in increased CR participation and maximizes its potential benefits for eligible patients (6). Indeed programs that feature early enrollment in home-based CR have been shown to improve functional status and quality of life, both of which are linked to improvements in long-term outcomes following CR (6, 25). In the present study we observed an average latency of 49 days between hospital discharge and enrollment in facility-based CR, with <25% of CBCR participants enrolling within 21 days following discharge, thus highlighting the need for novel interventions to improve these metrics.

### Limitations and Considerations

Our conclusions should be weighed against several limitations that warrant discussion, including the retrospective nature of our analysis of current CR utilization trends and our focus on one healthcare system (albeit one that encompasses members from many US states). More broadly, our conclusions regarding the potential clinical and economic benefits of HBCR are based on forecasts, which themselves make use of parameters (e.g., HBCR participation rate) were based on prior research involving MULTIFIT. All of these may limit the generalizability of our findings to other health systems and patient populations.

Prior studies have also shown challenges with HBCR adoption, given the large commitment and buy-in required from health systems, providers, and patients for a successful implementation (6). Additionally, given that HBCR is delivered via smartphone-based application, successful completion of HBCR requires a level of technological fluency that cannot be assumed among the older adults most typically referred for CR. In line with this concern, CR participation in our sample was higher among patients younger than age 60 compared to those aged 60+ years, who in turn had higher hospital readmission rates and consequent medical costs. This suggests that converting older patients—who have a higher risk of cardiac disease coupled with a potential hesitance or inability to attend in-person CR—into HBCR participants would likely contribute heavily to an economic savings realized through HBCR adoption; as an important counterpoint, however, in a population where CR participation was already high among older (vs. younger) adults, an economic analysis such as the one we conducted may not show as much potential benefit of HBCR implementation. Nonetheless, in contexts where participation is already low among older adults, overcoming technological barriers among this population will be an essential hurdle for HBCR programs. Our study can therefore serve as a template for other systems to independently assess the potential clinical and economic impact of implementing an HBCR program under realistic conditions.

Furthermore, our economic analysis utilized allowed amounts—the maximum possible cost to an insurer for medical services (e.g., hospital readmissions). Allowed amounts can diverge from actual paid amounts due to several idiosyncratic factors (e.g., variable hospital facility costs; patient insurance types; deductible progress throughout the year). Using allowed amounts to make economic calculations therefore achieves more consistency and is standard in the health insurance industry. To ensure that our results were interpretable, we used allowed amounts across the board in our economic analysis, including for facility-based CR, hospital readmissions, and Movn HBCR.

Finally, the per-patient price of $1,550 for delivery of HBCR is subject to change across populations and healthcare systems. We set this price *ad-hoc* for our economic analysis based on the current commercial price offered by Moving Analytics (rather than choosing a *post-hoc* price based on what would make our economic analysis appear favorable). Importantly, salaries for staff directly involved in HBCR administration (e.g., nurses; physicians) are bundled into this overall per-patient cost and facility costs are moot given the virtual nature of HBCR, all of which should limit variability in per-patient cost. Yet variability in future healthcare settings is still possible, due to fluctuations in costs related to identifying eligible patients and coordinating HBCR referrals, which fall outside of the per-patient cost used in our analysis. Future economic analyses could therefore yield somewhat different results from the ones presented above due to this variability.

## Conclusions

Our work suggests that there is significant opportunity to improve the CR implementation and delivery while generating substantial economic savings for third-party payers through adoption of a virtual, home-based CR program. We observed low participation rate for facility-based CR among eligible insured patients—a rate that could further dip during the ongoing COVID pandemic—and we projected significant cost savings from transitioning non-participants to HBCR and observing a subsequent reduction in hospital readmission rates. Introducing HBCR in a commercial payer environment may therefore prove to be beneficial to cardiovascular care more broadly by leading to improved outcomes among patients with CAD.

## Data Availability Statement

The raw data for this report was generated from insurance claims belonging to a private company and is therefore proprietary. Due to resulting privacy concerns, the data cannot be made publicly available. Further inquiries can be made to the corresponding author/s.

## Ethics Statement

The studies described in this report were reviewed and approved by the IRB at Highmark Health and informed consent was waived for the retrospective claims analysis. Written informed consent for participation was not required for this study in accordance with the national legislation and the institutional requirements.

## Author Contributions

AH and AW drafted the manuscript. Data were analyzed by two authors who are employed by Highmark Health (AW and KR). The sponsor and its affiliated authors (AH, AA, and HV) did not have access to the study data and participated in data interpretation only. All authors contributed to the article and approved the submitted version.

## Funding

Moving Analytics provided funding for the study through a contract with the VITAL Innovation Program at Highmark Health who facilitated the design and executed the analysis.

## Conflict of Interest

AH, AA, and HV report ownership interest in Moving Analytics. AA and HV are senior officers and receive salary support from Moving Analytics. The remaining authors declare that the research was conducted in the absence of any commercial or financial relationships that could be construed as a potential conflict of interest.

## Publisher's Note

All claims expressed in this article are solely those of the authors and do not necessarily represent those of their affiliated organizations, or those of the publisher, the editors and the reviewers. Any product that may be evaluated in this article, or claim that may be made by its manufacturer, is not guaranteed or endorsed by the publisher.
