# Supplementary material for: Retrospective Analysis and Forecasted Economic Impact of a Virtual Cardiac Rehabilitation Program in a Third-Party Payer Environment
Source: Front Digit Health. 2021 Nov 24;3:678009. doi: 10.3389/fdgth.2021.678009 (PMC8653769; doi:10.3389/fdgth.2021.678009)
Supplement: Supplementary file 1 [file Data_Sheet_1.DOCX]

**SUPPLEMENTARY MATERIALS**

**Supplementary Table S1.** Diagnosis and Procedural Codes Used

| Diagnosis Codes Used to Identify MI and CAD  **ICD-10 Codes** I20.1, I20.8, I20.9, I21.01, I21.02, I21.09, I21.11, I21.19, I2121, I2129, I21.3, I21.4, I21.9, I220, I221, I222, I228, I229, I21A1, I21A9, I23.7 I25.10, I25.111, I25.118, I25.119, I25.2, I25.41, I25.42, I25.5. I25.6, I25.701, I25.708, I25.709, I25.711, I25.721, I25.728, I25.729, I25.731, I25.738, I25.739, I25.791, I25.798, I25.799, I25.810, I25.82, I25.83, Z95.1, Z95.5, Z98.61 |
| --- |
| Diagnosis Codes Used to Identify PCI / Angioplasty / Other Revascularization  **CPT Codes**  92920, 92921, 92924, 92925, 92928, 92929, 92933, 92934, 92937, 92938, 92941, 92943, 92944, 92973  **HCPCS Codes**  C9601, C9602, C9603, C9604, C9605, C9606, C9607, C9608  **ICD 10 Codes**  0270046, 027004Z, 0270056,027005Z, 0270066, 027006Z, 0270076, 027007Z, 02700D6, 02700DZ, 02700E6, 02700EZ, 02700F6, 02700FZ, 02700G6, 02700GZ, 02700T6, 02700TZ, 02700Z6, 02700ZZ, 0270346,027034Z, 0270356, 027035Z, 0270366, 027036Z, 0270376, 027037Z, 02703D6, 02703DZ, 02703E6, 02703EZ, 02703F6, 02703FZ, 02703G6, 02703GZ, 02703T6, 02703TZ, 02703Z6, 02703ZZ, 0270446, 027044Z, 0270456, 027045Z, 0270466, 027046Z, 0270476, 027047Z, 02704D6, 02704DZ, 02704E6, 02704EZ, 02704F6, 02704FZ, 02704G6, 02704GZ, 02704T6, 02704TZ, 02704Z6, 02704ZZ, 0272046, 027204Z, 0272056, 027205Z, 0272066, 027206Z, 0272076, 027207Z, 02720D6, 02720DZ, 02720E6, 02720EZ, 02720F6, 02720FZ, 02720G6, 02720GZ, 02720T6, 02720TZ, 02720Z6, 02720ZZ, 0272346, 027234Z, 0272356, 027235Z, 0272366, 027236Z, 0272376, 027237Z, 02723D6, 02723DZ, 02723E6, 02723EZ, 02723F6, 02723FZ, 02723G6, 02723GZ, 02723T6, 02723TZ, 02723Z6, 02723ZZ, 0272446, 027244Z, 0272456, 027245Z, 0272466, 027246Z, 0272476, 027247Z, 02724D6, 02724DZ, 02724E6, 02724EZ, 02724F6, 02724FZ, 02724G6, 02724GZ, 02724T6, 02724TZ, 02724Z6, 02724ZZ, 0273046, 027304Z, 0273056, 027305Z,0273066, 027306Z, 0273076, 027307Z, 02730D6, 02730DZ, 02730E6, 02730EZ, 02730F6, 02730FZ, 02730G6, 02730GZ, 02730T6, 02730TZ, 02730Z6, 02730ZZ, 0273346, 027334Z, 0273356, 027335Z, 0273366, 027336Z, 0273376, 027337Z, 02733D6, 02733DZ, 02733E6, 02733EZ, 02733F6, 02733FZ, 02733G6, 02733GZ, 02733T6, 02733TZ, 02733Z6, 02733ZZ, 0273446, 027344Z, 0273456, 027345Z, 0273466, 027346Z, 0273476, 027347Z, 02734D6, 02734DZ, 02734E6, 02734EZ, 02734F6, 02734FZ, 02734G6, 02734GZ, 02734T6, 02734TZ, 02734Z6, 02734ZZ |
| Diagnosis Codes Used to Identify Coronary Artery Bypass Grafting (CABG)  **CPT Codes** 33510, 33511, 33512, 33513, 33514, 33516, 33517, 33518, 33519, 33521, 33522, 33523, 33530, 33533, 33534, 33535, 33536  **ICD 10 Codes** 0210083, 021088, 0210089, 021008C, 021008F, 021008W, 0210093, 0210098, 0210099, 021009C, 021009F, 021009W, 02100A, 02100A8, 02100A9, 02100AC, 02100AF, 02100AW, 02100J3, 02100J8, 02100J9, 02100JC, 02100JF, 02100JW, 02100K3, 02100K8, 02100K9, 02100KC, 02100KF, 02100KW, 02100Z3, 02100Z8, 02100Z9, 02100ZC, 02100ZF, 02100ZW, 0210344, 02103D4, 0210444, 0210483, 0210488, 0210489, 021048C, 021048F, 021048W, 0210493, 0210498, 0210499, 021049C, 021049F, 021049W, 02104A3, 02104A8, 02104A9, 02104AC, 02104AF, 02104AW, 02104D4, 02104J3, 02104J8, 02104J9, 02104JC, 02104JF, 02104JW, 02104K3, 02104K8, 02104K9, 02104KC, 02104KF, 2104KW, 02104Z3, 02104Z8, 02104Z9,02104ZC,02104ZF,02104ZW, 0211083, 0211088, 0211089, 21108C, 021108F, 021108W, 0211093, 0211098, 0211099, 021109C, 021109F, 021109W, 02110A3, 02110A8, 02110A9, 02110AC, 02110AF,02110AW, 02110J3, 02110J8, 02110J9, 02110JC, 02110JF, 02110JW, 02110K3, 02110K8, 02110K9, 02110KC, 02110KF, 02110KW, 02110Z3, 02110Z8, 02110Z9, 02110ZC, 02110ZF, 02110ZW, 0211344, 02113D4, 0211444, 0211483, 0211488, 0211489, 021148C, 021148F, 021148W, 0211493, 0211498, 0211499, 021149C, 021149F, 021149W, 02114A3, 02114A8, 02114A9, 02114AC, 02114AF, 02114AW, 02114D4, 02114J3, 02114J8, 02114J9, 02114JC, 02114JF, 02114JW, 02114K3, 02114K8, 02114K9, 02114KC, 02114KF, 02114KW, 02114Z3, 02114Z8, 02114Z9, 02114ZC, 02114ZF, 02114ZW, 0212083, 0212088, 0212089, 021208C, 021208F, 021208W, 0212093, 0212098, 0212099, 021209C, 021209F, 021209W, 02120A3, 02120A8, 02120A9, 02120AC, 02120AF, 02120AW, 02120J3, 02120J8, 02120J9, 02120JC, 02120JF, 02120JW, 02120K3, 02120K8, 02120K9, 02120KC, 02120KF, 02120KW, 02120Z3, 02120Z8, 02120Z9, 02120ZC, 02120ZF, 02120Z, 0212344, 02123D4, 0212444, 0212483, 0212488, 0212489, 021248C, 021248F, 021248W, 0212493, 0212498, 021249, 021249C, 021249F,021249W, 02124A3, 02124A8, 02124A9, 02124AC, 02124AF, 02124AW, 02124D4, 02124J3, 02124J8, 02124J9, 02124JC, 02124JF, 02124JW, 02124K3, 02124K8, 02124K9, 02124KC, 02124KF, 02124KW, 02124Z3, 02124Z8, 02124Z9, 02124ZC, 02124ZF, 02124ZW, 0213083, 0213088, 0213089, 021308C, 021308F, 021308W, 0213093, 0213098, 0213099, 021309C, 021309F, 021309W, 02130A3, 02130A8, 02130A9, 02130A, 02130AF, 02130AW, 02130J3, 02130J8, 02130J9, 02130JC, 02130JF, 02130JW, 02130K3, 02130K8, 02130K9, 02130KC, 02130KF, 02130KW, 02130Z3, 02130Z8, 02130Z9, 02130ZC, 02130ZF, 02130ZW, 0213344,02133D4, 0213444, 0213483, 0213488, 0213489, 021348C, 021348F, 021348W, 0213493, 0213498, 0213499, 021349C, 021349F, 021349W, 02134A3, 02134A8, 02134A9, 02134AC, 02134AF, 02134AW, 02134D4, 02134J3, 02134J8, 02134J9, 02134JC, 02134JF, 02134JW, 02134K3, 02134K8, 02134K9, 02134KC, 02134KF, 02134KW, 02134Z3, 02134Z8, 02134Z9, 02134ZC, 02134ZF, 02134ZW |
| Codes Used to Identify Cardiac Rehab Participation  **CPT Codes**  93797, 93798, 93799 |

**Supplementary Table S2.** Cardiac Rehabilitation Eligibility and Participation Stratified by Age and Gender

| **Gender** | **Age  (years)** | **CR Eligible Members (N)** | **CR Eligible Members with  CR Claim (N)** | **CR Eligible Members with  CR Claim (%)** |
| --- | --- | --- | --- | --- |
| Female | ≤30 | 2 | 0 | 0% |
|  | 31–40 | 34 | 12 | 35% |
|  | 41–50 | 156 | 65 | 42% |
|  | 51–60 | 406 | 149 | 37% |
|  | 61–70 | 527 | 215 | 41% |
|  | 71–80 | 638 | 181 | 28% |
|  | 81–90 | 502 | 72 | 14% |
|  | >90 | 124 | 1 | 1% |
|  | All Females | 2,389 | 695 | 29% |
| Male | ≤30 | 10 | 1 | 10% |
|  | 31–40 | 68 | 27 | 40% |
|  | 41–50 | 454 | 173 | 38% |
|  | 51–60 | 1259 | 585 | 46% |
|  | 61–70 | 1434 | 641 | 45% |
|  | 71–80 | 1041 | 409 | 39% |
|  | 81–90 | 534 | 129 | 24% |
|  | >90 | 75 | 3 | 4% |
|  | All Males | 4,875 | 1,968 | 40% |
| Values are range of age in years, N, or % of N. CR = cardiac rehabilitation | | | | |

**Supplementary Table S3.** Cardiac Rehabilitation Eligibility and Enrollment Stratified by Coverage

| **Age** | **CR Eligible Members** | **CR Eligible Members with CR Claim (*N*)** | **CR Eligible Members with  CR Claim (%)** |
| --- | --- | --- | --- |
| Commercial | 3,991 | 1,657 | 42% |
| Senior-oriented  (i.e., Medicare, Medicaid, Medigap) | 3,144 | 965 | 31% |
| Coverage not listed | 129 | 41 | 32% |
| Values are N or % of N. CR = cardiac rehabilitation | | | |

**Supplementary Table S4.** Top 20 Hospital Readmission Etiologies Among Eligible Patients**.**

| **Primary Diagnosis** | **Description** |
| --- | --- |
| I214 | Non-ST elevation (N-STEMI) myocardial infarction |
| I130 | Hypertensive heart and chronic kidney disease with heart failure and Stage 1 through Stage 4 chronic kidney disease, or unspecified chronic kidney disease |
| I25110 | Atherosclerotic heart disease of native coronary artery with unstable angina pectoris |
| I2510 | Atherosclerotic heart disease of native coronary artery without angina pectoris |
| I110 | Hypertensive heart disease with heart failure |
| A419 | Sepsis, unspecified organism |
| N179 | Acute kidney failure, unspecified |
| J189 | Pneumonia, unspecified organism |
| I350 | Nonrheumatic aortic (valve) stenosis |
| J441 | Chronic obstructive pulmonary disease with (acute) exacerbation |
| I4891 | Unspecified atrial fibrillation |
| N390 | Urinary tract infection |
| I25119 | Atherosclerotic heart disease of native coronary artery with unspecified angina pectoris |
| I472 | Ventricular tachycardia |
| I480 | Paroxysmal atrial fibrillation |
| K992 | Gastrointestinal hemorrhage |
| I5023 | Acute on chronic systolic (congestive) heart failure |
| I639 | Cerebral infarction |
| J9601 | Acute respiratory failure with hypoxia |
